# Making the invisible visible: New perspectives on the intersection of human–environment interactions of clinical teams in intensive care

**DOI:** 10.1038/s41372-021-01160-0

**Published:** 2021-08-21

**Authors:** Sheena Visram, Laura Potts, Neil J. Sebire, Yvonne Rogers, Emma Broughton, Linda Chigaru, Pratheeban Nambyiah

**Affiliations:** 1 Department of Computer Science/UCL Interaction Centre, University College London, London, UK; 2 Digital Research, Informatics and Virtual Environments (DRIVE) Centre, Great Ormond Street Hospital for Children, London, UK; 3 Clinical Simulation Centre, Great Ormond Street Hospital for Children, London, UK

**Keywords:** Paediatrics, Scientific community

## Abstract

Understanding human behaviour is essential to the successful adoption of new technologies, and for the promotion of safer care. This requires capturing the detail of clinical workflows to inform the design of new human–technology interactions. We are interested particularly in the possibilities for touchless technologies that can decipher human speech, gesture and motion and allow for interactions that are free of contact. Here, we employ a new approach by installing a single 360° camera into a clinical environment to analyse touch patterns and human–environment interactions across a clinical team to recommend design considerations for new technologies with the potential to reduce avoidable touch.

There is increasing interest in how healthcare workers interact with technology and the cognitive processes that underpin these interactions. One example is gesture-based control of image manipulation within medical scans [1], but many other healthcare settings involve complex human/technology interactions. While medical devices and information technologies continue to improve outcomes, their proliferation has reduced standardisation compared to other safety-critical industries, which has implications for team performance and potentially patient outcomes.

Human factors and ergonomic (HFE) principles arose from endeavours to improve the safety of military and transport systems and are also important in understanding human–environment interactions in healthcare [2]. Our focus is on human/technology interactions within the neonatal intensive care unit (NICU) [3], wherever more sophisticated technology is regularly introduced.

Clinical simulation offers an ideal approach to examine interactions in an acute clinical scenario in a safe and controlled setting. The scenario can be observed using an unobtrusive 360° camera and viewed on a 360° media player using a virtual reality (VR) headset. The remote but immersive observation of a clinical team managing a high-fidelity simulation of a deteriorating preterm infant provides a new perspective to consider optimum conditions for the touchless technologies with the potential to reduce avoidable touch, whilst minimising the effect of an observer’s presence.

A clinical team (three nurses, one doctor; all-female) participated in a simulated scenario lasting 17 min. Analysis revealed 437 episodes and 68 sequence pairs of touch across 17 surfaces. Touch patterns differed across the clinical team, who worked in pairs as a safety system to identify, investigate, manage and record the clinical deterioration. Assessment of vital signs, clinical examination, and equipment for ultrasound and transillumination was contained in a restricted incubator space, interrupted by retrieval of equipment requiring assembly and disassembly and diversions of focus to multiple in-room monitors. At a second focal point, medication preparation involves hand-offs of medications and ancillaries and manual initiation of infusion pumps.

We developed a coding system comprising sequences and episodes of touch enabling classifications of human–environment interactions. This included recording touch frequency by a single touch, sequential touch incidents and patterns of touch across individual team members. From this, we developed a classification of avoidable touch during tasks. Four types of potentially avoidable touch were analysed: ‘repeated touch’, ‘obstructions to touch’ ‘touch that cause delays’ and ‘unplanned touch’. Unavoidable touch for care provision was also identified.

This preliminary study of a high-fidelity simulation representative of a clinical environment characterised by complex and frequent flows of decisions focused on the translation of hand touch patterns across a clinical team. Detailed touch analysis of a simulated clinical scenario enabled exploration of the implications of different configurations of touchless technologies were introduced, with potential positive impacts on efficiency, effectiveness, and potential for infection transmission [4]. This study also highlighted the importance of understanding team behaviours in terms of interactions that make up clinical workflows, as well as traditional ergonomic aspects of the environment, such as effective use of space, suitable lighting, minimising obstructions and limiting ambient noise to improve integration as summarised in Fig. 1. It suggests that new technology must also match the reliability of traditional methods and add value to the clinical team as part of a clinical workflow.

**Fig. 1:**
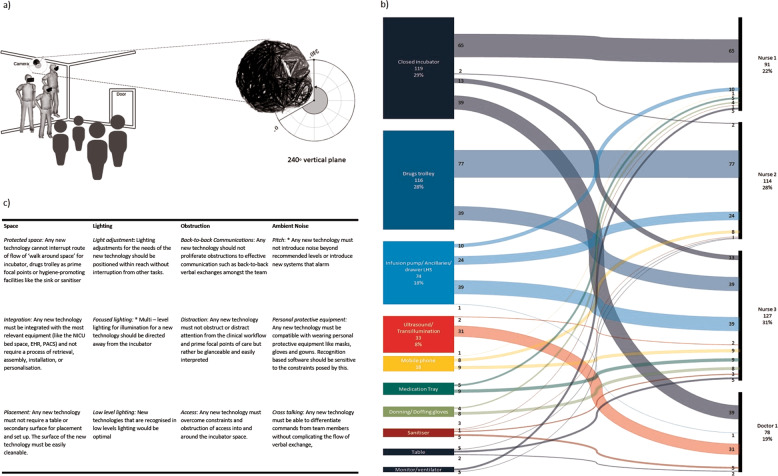
Human-technology interactions in NICU. (**a**) Aerial placement of a 360° camera in NICU to emulate the viewpoint of an observer, to conduct an interaction analysis including touch frequency, as illustrated by (**b**) Sankey chart of the top 10 most commonly touched equipment by the individual, accounting for 410 of the episodes of touch, and thereafter to infer (**c**) optimal conditions for touchless technologies by space, lighting, obstructions, and ambient noise in NICU (SLOAN framework) EHR refers to Electronic Health Record and PACS refers to Picture archiving and communications system. *Derived from White [5].

Touch forms the basis of the relationship between healthcare workers, their tasks, the tools they use, and the physical environment they work in. This analysis of touch patterns offers new insight into the working of clinical teams using an unobtrusive 360° camera to capture and enable rich touch pattern analysis. It provides a potential paradigm for future evaluations of healthcare environments when assessing the potential of new technologies for healthcare.
